# A Flow Cytometric Analysis of the Inhibition of Platelet Reactivity Due to Nitrite Reduction by Deoxygenated Erythrocytes

**DOI:** 10.1371/journal.pone.0092435

**Published:** 2014-03-18

**Authors:** Krittapoom Akrawinthawong, Ji Won Park, Barbora Piknova, Nathawut Sibmooh, Suthat Fucharoen, Alan N. Schechter

**Affiliations:** 1 Molecular Medicine Branch, National Institutes of Diabetes and Digestive and Kidney Diseases, National Institutes of Health, Bethesda, Maryland, United States of America; 2 Department of Pharmacology, Faculty of Science, Mahidol University, Bangkok, Thailand; 3 Department of Biochemistry, Faculty of Medicine, Siriraj Hospital, Mahidol University, Bangkok, Thailand; 4 Thalassemia Research Center, Institute of Science and Technology for Research and Development, Mahidol University, Nakhonpathom, Thailand; Albany Medical College, United States of America

## Abstract

Nitric oxide (NO), a small gas molecule, has long been known to be a potent inhibitor of platelet function but the physiological and pathological implications of platelet inhibition by NO have not been well clarified. We recently showed that the addition of nitrite to platelet-rich plasma in the presence of erythrocytes could inhibit platelet aggregation and this inhibitory effect of nitrite + erythrocytes was enhanced by deoxygenation of erythrocytes as measured by P-selectin expression and cGMP production. In order to study the nitrite effect on platelets at different oxygen levels, we used the flow cytometric assays to detect platelet membrane surface markers upon activation. The P-selectin and activated gpIIb/IIIa expression on platelet membranes in response to ADP, collagen and thrombin stimulation was measured at various hematocrit and oxygen levels. Nitrite (0.1 to 1.0 μM) significantly decreased the percentage of these surface markers on the platelet membrane at the hematocrit values above 23% and oxygen levels lower than 49 mmHg. The inhibitory effect of nitrite was augmented by increasing hematocrit values and decreasing oxygen saturation. C-PTIO (an NO scavenger) prevented the platelet inhibition by nitrite + erythrocytes whereas the inhibitors of NO synthase and xanthine oxidoreductase had no effect. These results support the proposal that circulating nitrite decreases platelet reactivity in the presence of partially deoxygenated erythrocytes through its reduction to NO, which may also explain certain differences between arterial and venous thrombosis and support directly the role of deoxyhemoglobin in this process. We believe that our flow cytometric assays offer a possibility to identify the individual molecular process involved in these effects.

## Introduction

Platelets are non-nucleated cell fragments derived from megakaryocytes and are highly reactive entities that contribute to the coagulation processes by binding to injured vessel walls and forming aggregates with other platelets. Platelet activation can occur by various stimuli released from the endothelium and blood cells themselves. Numerous agonists work on platelet membrane receptors which perceive and transduce activation signals into downstream pathways [1]. For example, both adenosine diphosphate (ADP) and thrombin work on G-protein coupled receptors (GPCRs), which are P2Y and protease-activated receptors (PARs) respectively [2]. In addition, collagen, one of the major components in the vessel wall, can act as a signal when exposed at the site of injury because platelets start to adhere to the damaged vessel wall and interact with other platelets through their glycoprotein (GPVI) and integrin (α2β1) receptors [3]. Therefore, it is critical to maintain a tight balance between pro- and anti-thrombotic signals within the circulation to precisely regulate platelet function and keep normal vascular flow.

Nitric oxide (NO) is known to be a key molecule playing roles in the regulation of vascular homeostasis through platelet inhibition and vasodilation effects. The inhibitory effect of NO on platelet activation is thought to be mainly through cyclic guanosine 3′,5′-monophosphate (cGMP)-dependent pathways resulting in decreased phosphorylation of downstream proteins, platelet degranulation, and intra-platelet calcium mobilization [4]. Most NO in the vascular system is synthesized by nitric oxide synthase (NOS) which has been recognized as a key enzyme. The endothelial NOS (eNOS)-derived NO is well known to inhibit platelet adhesion and aggregation [5], [6]. However, an alternative pathway for NO generation was recently established in which the reduction of nitrate (NO_3_^−^) and nitrite (NO_2_^−^) to NO by non-enzymatic as well as enzymatic pathways are involved. It has been shown that nitrate absorbed from digestive tract can be reduced to nitrite by bacterial nitrate reductases in the oral cavity [7]. Nitrite can be further reduced to NO by several pathways including deoxyhemoglobin [8], [9], deoxymyoglobin [10], xanthine oxidase [11], [12], and non-enzymatic reduction in the presence of protons [13], [14] or ascorbic acid [15]. Since nitrate and nitrite can be easily obtained from our diet, the potential NO-related bioactivities of those anions are getting more attention with regard to the cardiovascular benefits [16], [17]. We previously reported that nitrite could inhibit platelet aggregation and activation in the presence of erythrocytes through its reduction to NO and this inhibitory effect was promoted by deoxygenation since deoxyhemoglobin reduces nitrite to NO [18]. Furthermore, nitrate ingestion in the form of beetroot juice has been recently shown to have anti-platelet activity in healthy volunteers [19]. This suggests that nitrite might play a critical role in regulating platelet reactivity under hypoxic conditions.

Since nitrite has a potential effect on platelets, it is important to study how nitrite plays a role in inhibiting platelet activation pathways and how this would affect overall dynamic processes of the blood clotting systems. In the current study, we analyzed the effect of nitrite on two different platelet activation pathways by monitoring membrane markers, P-selectin and glycoprotein IIb/IIIa (gpIIb/IIIa). P-selectin is secreted from the alpha granules of platelets and translocated to the membrane upon activation and mediates stable adhesions between cells [20]. The fibrinogen receptor, gpIIb/IIIa, undergoes conformational changes upon platelet activation, which allows fibrinogen to bind to gpIIb/IIIa, resulting in platelet aggregation [21]. We showed here that nitrite inhibited P-selectin expression on the platelet membrane and gpIIb/IIIa activation in response to ADP, collagen and thrombin stimulation in the presence of erythrocytes and this inhibition was promoted by increasing hematocrit and deoxygenation of erythrocytes, suggesting that NO produced by the reaction of nitrite with deoxyhemoglobin was responsible for this inhibitory effect.

## Materials and Methods

### Ethics statement

This study was approved by NIH ethics committee (protocol#99-CC-0168). The written informed consent was obtained in accordance with the declaration of Helsinki.

### Blood sample

Healthy volunteers who were not on any medication at least for 2 weeks were randomly recruited through National Institutes of Health (NIH) blood bank. Female to male ratio was 25 to 17 with age of 37.2±5.2 years. Blood was collected and immediately mixed with 3.8% sodium citrate (9∶1). Blood samples were used for experiments within 3 hours after withdrawal.

### Materials

Sodium nitrite (NaNO_2_), adenosine 5′ diphosphate (ADP), collagen, human thrombin, N^G^-Nitro-L-Arginine Methyl Ester (L-NAME), Gly-Pro-Arg-Pro (GPRP), oxypurinol and paraformaldehyde were purchased from Sigma (St.Louis, MO). Diethylamine diazeniumdiolate (DEANONOate) was purchased from Cayman chemical (Ann Arbor, MI), 2-(4-carboxyphenyl)-4,4,5,5-tetramethylimidazole-1-oxyl 3-oxide (C-PTIO) was purchased from Alexis (Lausen, Switzerland). FITC-labeled PAC1, PE-labeled anti-human CD62P, PE-Cy5 anti-human CD41a and FITC- or PE-conjugated isotypic MoAbs, and flow cytometry graded PBS were purchased from Becton Dickinson (BD; San Jose, CA).

### Sample preparation

#### Preparation of platelet rich plasma (PRP) and erythrocytes

Whole blood was centrifuged at 120 *g* for 10 minutes at room temperature (25°C). Platelet rich plasma (PRP) in the upper portion was collected gently and the lower portion was centrifuged further at 2000 *g* for another 20 minutes. Then, plasma and buffy coat in the upper portion were removed and packed erythrocytes in lower portion were collected. Hematocrit (Hct) was adjusted by adding erythrocytes into PRP per volume calculation. Then, Hct level was confirmed by conventional method of capillary tube spinning with Hct graph. Also, the concentration of platelets per volume was adjusted to achieve equal exposure to platelet agonists by flow cytometry.

Incubation of NO donors or nitrite with PRP or PRP+erythrocytes was done at 37°C for 5 minutes. For platelet activation, ADP (20 μM), collagen (20 μg/mL) and thrombin (1 U/mL) were used to achieve the maximal expression of platelet markers (P-selectin & activated GpIIb/IIIa). For thrombin, GPRP (peptide Gly-Pro-Arg-Pro) was added to prevent fibrin clot formation.

#### Adjustment and measurement of oxygen concentration

Deoxygenation of erythrocytes was done by helium gas blown above the cell suspension with gentle stirring. The deoxygenated erythrocytes were stored in a vacuum container until use. For oxygenation, oxygen was used instead of helium. Oxygen concentration and free hemoglobin were measured by ABL-80 FLEX Co-ox blood gas analyzer (Radiometer America Inc.).

### Flow cytometry

Each monoclonal antibody (MoAb) was added to the cell suspension and the mixture was incubated at room temperature for 15 minutes in the dark. Then, the mixture was fixed with 1% paraformaldehyde to prevent further activation of platelets. Samples were diluted with PBS and analyzed with FACS Calibur flow cytometry (BD, Oxford, UK) at 488 nm wavelength. Platelets were identified by positive PE-Cy5 fluorescence. The percentage of PAC1-FITC or CD62P-PE positive cells was determined by analyzing 10,000 platelet specific signals for PE-Cy5 fluorescence. Acquisition rate was limited at 1,000 platelets/sec to prevent coincidental detection of more than one particle.

### Statistical analysis

Statistical analysis was performed with Statistical Package for the Social Science (SPSS) program (version 15.0) and data are mean ± SEM. ANOVA with Tukey's method was used with p-value <0.05. All graphs were constructed by GraphPad Prism version 5.

## Results

### Nitrite inhibits platelet activation in the presence of erythrocytes

**Figure 1:**
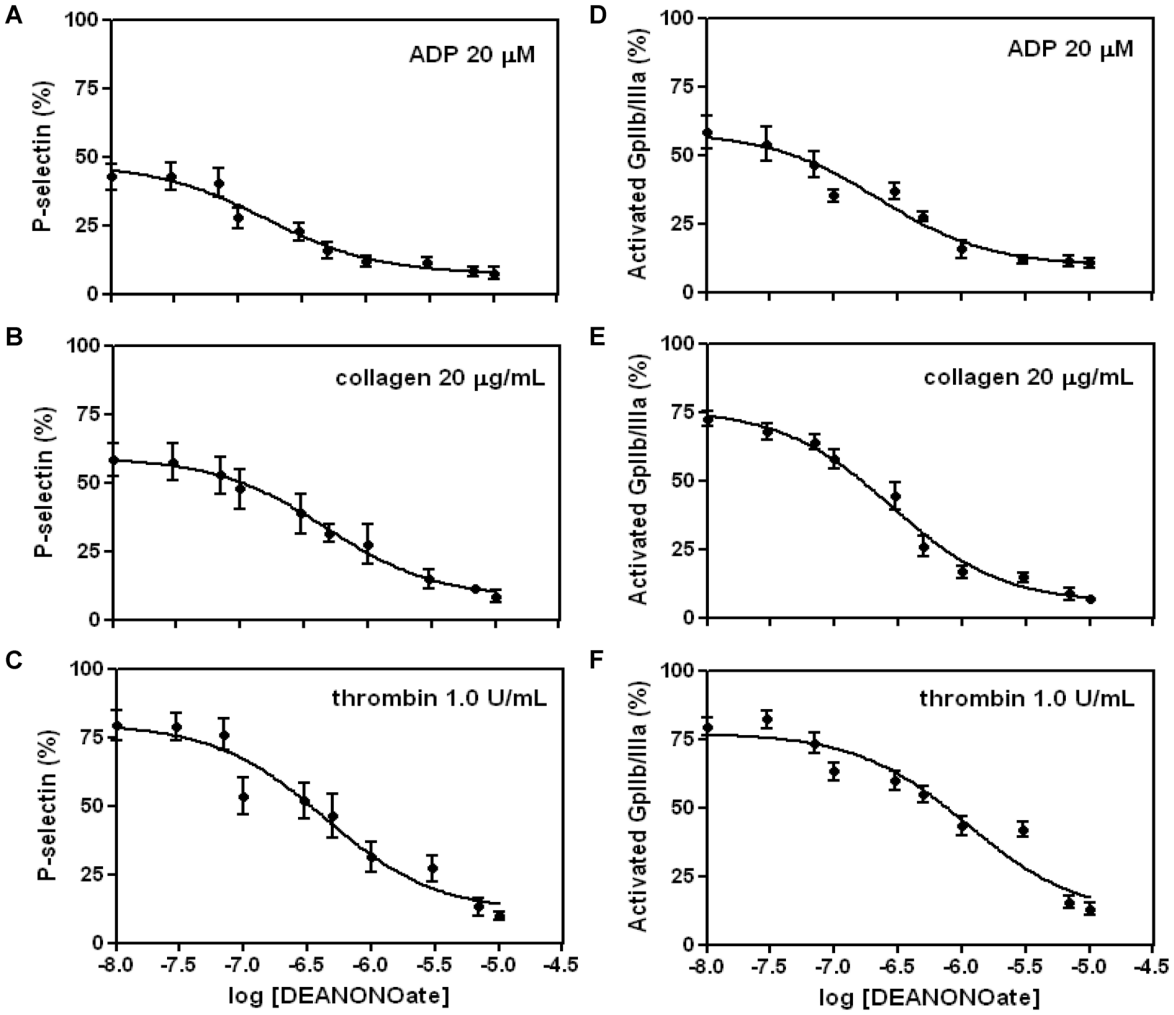
Effect of DEANONOate on platelet activation in platelet-rich plasma + erythrocytes. Platelet activation was induced by ADP (A, D, 20 μM), collagen (B, E, 20 μg/mL) and thrombin (C, F, 1 U/mL) in platelet-rich plasma + erythrocytes preincubated with DEANONOate at 37°C for 2 minutes. Platelet activation was analyzed by measuring the percentage of cells expressing P-selectin (A, B, C) and activated gpIIb/IIIa (D, E, F) on the membrane using specific antibodies (CD62P and PAC-1, respectively, mean Hct 40%, mean pO_2_ 49 mmHg, n = 6).

**Figure 2:**
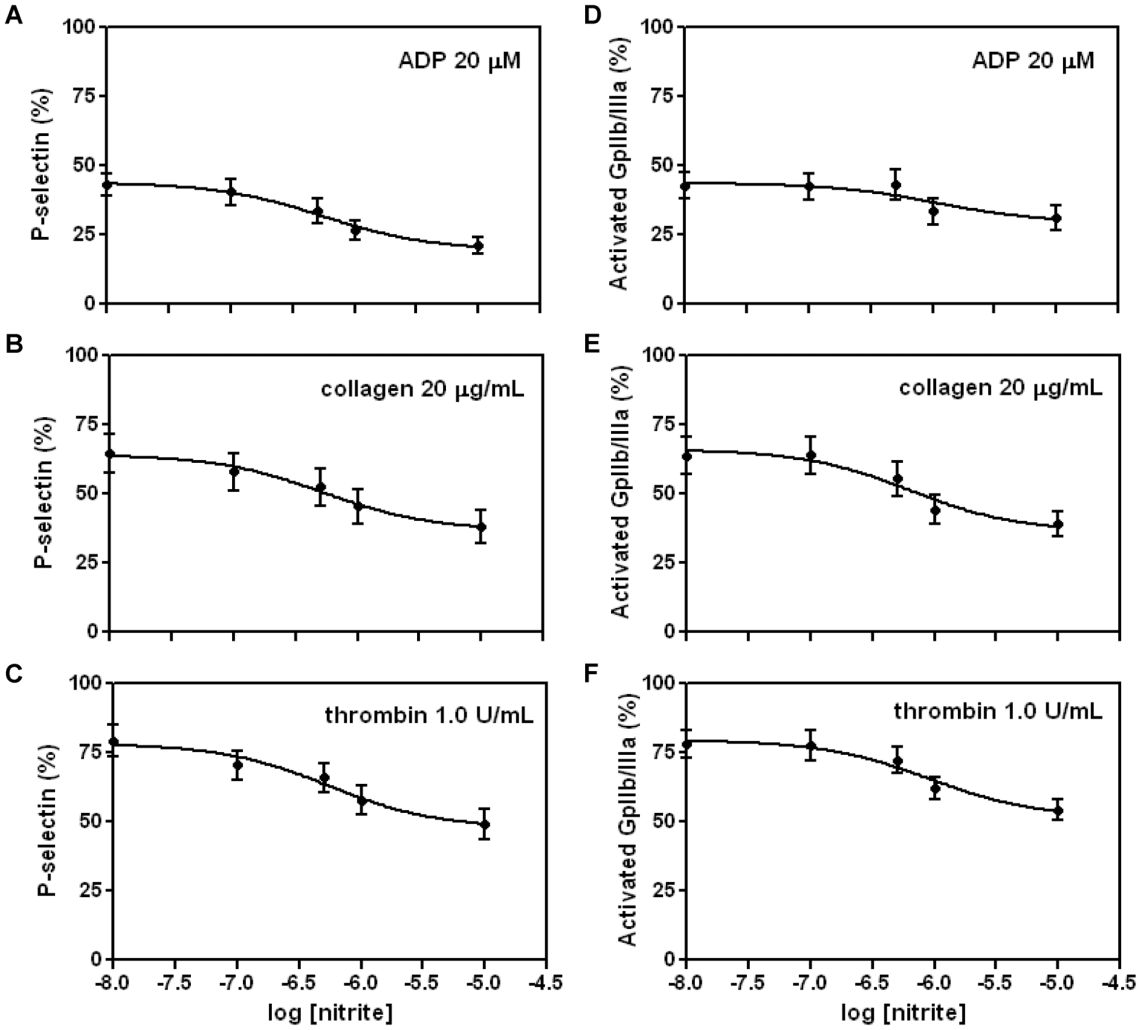
Effect of nitrite on platelet activation in platelet-rich plasma + erythrocytes. Platelet-rich plasma + erythrocytes were preincubated with nitrite (1 μM) at 37°C for 5 minutes and platelet activation was induced by ADP (A, D, 20 μM), collagen (B, D, 20 μg/mL) and thrombin (C, F, 1 U/mL). P-selectin (A, B, C) and activated gpIIb/IIIa expression (D, E, F) was monitored. (CD62P and PAC-1, respectively, mean Hct 40%, mean pO_2_ 49 mmHg, n = 6).

Varied concentrations of DEANONOate (half-life of 2 min) and nitrite (0.01–10 μM) were used to examine the inhibitory effect of these potential NO donors on platelet activation measured by the percentage of P-selectin or activated gpIIb/IIIa positive cells (Fig. 1 and 2). Platelet activation was induced by three agonists, ADP (20 μM), collagen (20 μg/ml), and thrombin (1 U/ml). DEANONOate showed a clear inhibitory effect for both P-selectin and activated gpIIb/IIIa expression with the log IC_50_ from −5.96 to −6.78 in platelet-rich plasma + erythrocytes (mean Hct 40%, mean pO_2_ 49 mmHg, Fig. 1). Nitrite also showed a similar inhibition pattern to DEANONOate although the total extent of inhibition was less with nitrite than with DEANONOate (Fig. 2). We found that log IC_50_ of nitrite concentration (−5.95 to −6.27) was similar to that of DEANONOate concentration (−5.96 to −6.78). Thus, nitrite at 1 μM was chosen for further experiments. However, nitrite showed no effect on thrombin-, ADP- and collagen-induced platelet activation when measured in platelet rich plasma (data not shown).

### Platelet inhibition by nitrite + erythrocytes is promoted by deoxygenation

**Figure 3:**
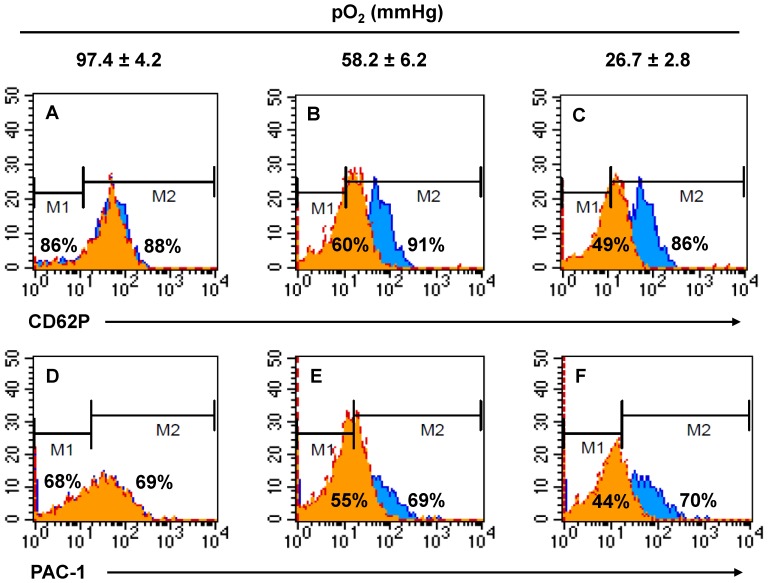
Nitrite inhibits thrombin-induced platelet activation in the presence of deoxygenated erythrocytes. Representative flow diagrams demonstrate the inhibitory effects of nitrite on the thrombin-induced activation of platelets. Platelet activation was analyzed by monitoring the percentage of cells expressing P-selectin (A, B, C) and activated gpIIb/IIIa (D, E, F) after thrombin (1 U/mL) stimulation in both control (blue) and nitrite (1 μM; orange) treated group in the presence of erythrocytes (Hct 42.6%) with different oxygen concentrations [pO_2_; 97.4±4.2 mmHg (A,D), 58.2±6.2 mmHg (B,E) and 26.7±2.8 mmHg (C,F), n = 6].

Next, we investigated whether the effects of nitrite on platelet activation would be dependent on the oxygenation state of erythrocytes assuming these effects are mediated by deoxyhemoglobin through the reduction of nitrite to NO. We added erythrocytes which were prepared to have three different oxygen levels, to the control or nitrite-preincubated platelet-rich plasma and then measured the expression of P-selectin and activated gpIIb/IIIa after thrombin stimulation. At physiological hematocrit (42.6%), the expression of P-selectin and activated gpIIb/IIIa decreased greatly when deoxygenated erythrocytes (pO_2_ 58.2±6.2 mmHg) were added to nitrite-preincubated platelet-rich plasma compared to when fully oxygenated erythrocytes (pO_2_ 97.4±4.2 mmHg) were added (P-selectin:86 to 60%, gpIIb/IIIa:68 to 55%, Fig. 3. orange histogram). This inhibitory effect of nitrite was even more pronounced when severely deoxygenated erythrocytes (26.7±2.8 mmHg) were added (P-selectin:86 to 49%, gpIIb/IIIa:68 to 44%). However, the addition of erythrocytes with different oxygen levels did not affect P-selectin exposure and gpIIb/IIIa activation in the absence of nitrite (Fig. 3. blue histogram)

**Figure 4:**
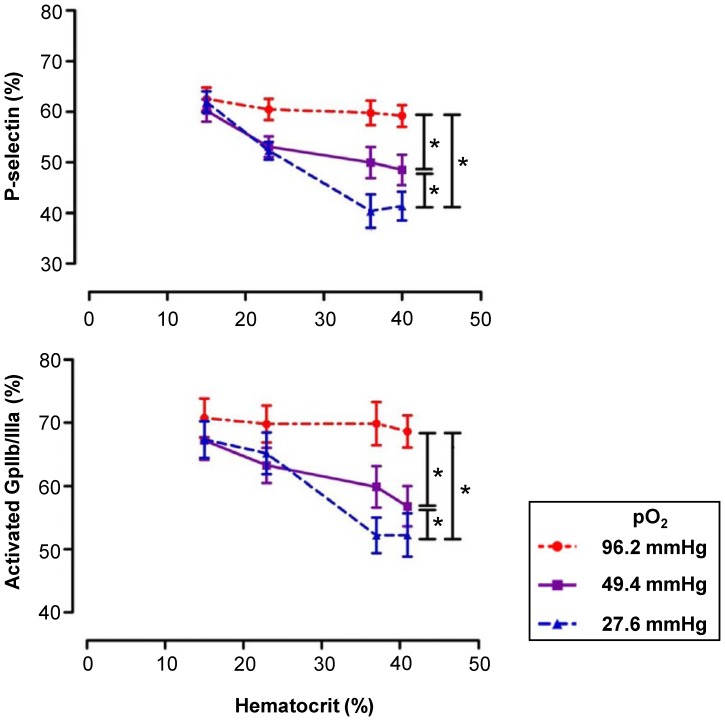
Effect of hematocrit changes on the thrombin-induced platelet activation in platelet-rich plasma + erythrocytes. Nitrite (1 μM) was added to platelet-rich plasma + erythrocytes with various hematocrits (15.4±1.8, 23.0±2.3, 36.7±1.6, 40.2±1.2%) before thrombin (1 U/mL) stimulation and the percentage of cells expressing P-selectin and activated gpIIb/IIIa were measured (n = 6, * p<0.05).

We then examined whether the inhibitory effects of nitrite would be affected by changes of hematocrit (Fig. 4). We started to observe the nitrite effect in decreasing the expression of P-selectin and activated gpIIb/IIIa at 23% hematocrit only when erythrocytes were deoxygenated, and this effect further increased at higher hematocrit (37∼40%). Consistent with the results from Figure 3, the more we decreased oxygen levels of erythrocytes, the more platelets were inhibited by nitrite at physiological hematocrit levels.

**Figure 5:**
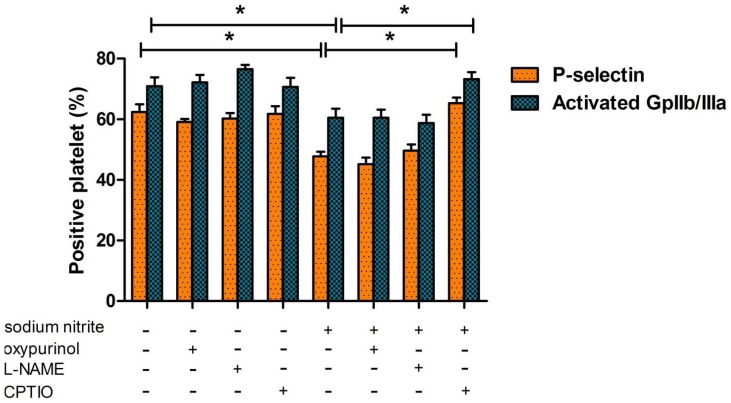
Platelet inhibition by nitrite is mediated by its reduction to NO. NO scavenger (CPTIO) or enzyme inhibitors (L-NAME for NOS and oxypurinol for XOR) were preincubated at 37°C for 5 minutes with platelet-rich plasma + erythrocytes before nitrite addition and then thrombin-induced P-selectin exposure and gpIIb/IIIa activation were analyzed (mean Hct 43.4%, pO_2_ 55.4 mmHg, thrombin 1 U/mL, nitrite 1 μM, oxypurinol 100 μM, L-NAME 300 μM, CPTIO 200 μM, n = 6, * p<0.05).

To test the involvement of enzymatic pathways in nitrite inhibition of platelet activation, we used oxypurinol (100 μM, a xanthine oxidoreductase (XOR) inhibitor) and L-NAME (300 μM, a NOS inhibitor) (Fig. 5). We observed no significant differences in nitrite effects on P-selectin and activated gpIIb/IIIa expression when we preincubated platelet-rich plasma+erythrocytes with oxypurinol or L-NAME. However, preincubation with C-PTIO (200 μM, a membrane-impermeable NO scavenger) completely prevented nitrite effect on platelet activation in platelet-rich plasma+erythrocytes suggesting that the inhibitory effect of nitrite was mediated by its reduction to NO, not by endogenous XOR or NOS pathways.

## Discussion

Regulation of platelet function in the vasculature is a tightly-controlled process. Since NO is known to be a potent inhibitor of platelet function, it is important to understand how NO orchestrates complex and dynamic platelet physiology and contributes to vascular homeostasis. Although the effects of endothelium-derived NO on platelets have been reported since 1980's [5], [22], [23], there was little interest in the potential effects of nitrite on platelet function at that time. However, since it has been established that NO can be generated by the stepwise reduction of nitrate through nitrite [24], the therapeutic potentials of nitrite and nitrate are getting increased attention for their roles in preventing and treating cardiovascular diseases [17]. Our previous report showed the inhibitory effects of nitrite on human platelet aggregation and granule secretion as well as cGMP production in the presence of erythrocytes and this was abolished by an NO scavenger, which suggests that nitrite inhibits platelet functions through its reduction to NO mediated by partially deoxygenated erythrocytes [18]. We also developed in the same study a flow cytometric method to detect P-selectin exposure in response to ADP stimulation as an efficient tool to evaluate nitrite effect on platelet activation. In the current study, we extended this method to several agonists and markers under various experimental conditions to demonstrate that nitrite inhibits major platelet activation pathways involving granule secretion and fibrinogen receptor activation only in the presence of deoxygenated erythrocytes, which is of physiological importance under hypoxic and ischemic conditions. We monitored the effect of nitrite on two platelet activation pathways using flow-cytometric analysis of P-selectin exposure to the platelet membrane and conformational change of the fibrinogen receptor, gpIIb/IIIa, on the membrane upon agonist stimulation. Consistent with our and other previous reports [18], [25], we also confirmed that nitrite at physiological concentrations did not show any effect on P-selectin and activated gpIIb/IIIa levels in platelet-rich plasma, while the NO donor, DEANONOate clearly exhibited an inhibition of P-selectin and activated gpIIb/IIIa levels in platelet-rich plasma (data not shown). However, nitrite inhibited platelet activation after ADP, collagen or thrombin stimulation when platelet- rich plasma was incubated with deoxygenated erythrocytes (Figure 2) although the degree of inhibition was not as great as that of DEANONOate (Figure 1). It seems that this nitrite effect was mediated by its reduction to NO since we showed that NO scavenger, CPTIO, abolished this inhibitory effect of nitrite, while NOS or XOR inhibition did not affect nitrite effects on platelet reactivities (Figure 5). It is expected that the endogenous NOS pathway becomes inactive under hypoxic conditions because the NOS reaction requires oxygen; in contrast, nitrite and nitrate reduction pathways are enhanced under hypoxic conditions and this makes the reductive pathways relevant backup systems for NO generation. Since our diet is usually rich in vegetables which are a very good source for nitrate, it provides enough substrates to the symbiotic bacteria in the oral cavity, which are responsible for the initial reduction of nitrate into nitrite [7]. Then, nitrite can be further reduced to NO by numerous mechanisms as listed in the introduction.

In our study, platelet inhibition by nitrite is very likely to be mediated by the deoxyhemoglobin which is present in deoxygenated erythrocytes. We propose that deoxyhemoglobin reacts with nitrite near the cytoplasmic surface of erythrocyte membrane forming iron-nitrosyl-hemoglobin (HbNO) and NO can be released from HbNO by cellular oxidants or ascorbic acid metabolites [26], [27]. When we used two different levels of deoxygenation, we showed that the inhibitory effect of nitrite was augmented with increasing deoxygenation levels, while nitrite did not show any effect in platelet-rich plasma with fully oxygenated erythrocytes (Figure 3 and 4). These results support the idea that nitrite might be a preferential source for NO under hypoxic situations *in vivo*. Our results also show that increasing hematocrit enhances the inhibitory effect of nitrite on platelet activation (Figure 4). We observed an inhibitory effect of nitrite on P-selectin and activated gpIIb/IIIa levels from approximately 20% hematocrit when partially or severely deoxygenated erythrocytes were added to platelet-rich plasma and this effect was further promoted at higher hematocrit (∼40%). These results provide evidences that the amount of deoxyhemoglobin available in the vasculature could determine the degree of nitrite inhibition of platelet activation. Our results are consistent with previous reports that inorganic nitrate ingestion inhibited human platelet aggregation [28] and oral intake of nitrate-rich beetroot juice decreased platelet aggregation [29] although those studies did not provide mechanistic insights on how nitrate-nitrite-NO pathways could contribute to regulation of platelet activities. We also demonstrated in our recent report that the levels of blood nitrite and nitrate substantially affect tail bleeding time in mice. Nitrite supplementation prolonged bleeding time compared with control, in contrast, low nitrite/nitrate diet shortened bleeding time [30]. These results suggest that nitrite levels *in vivo* might be a critical factor for regulating platelet reactivity.

In our current study, we demonstrate that nitrite plays a significant role in inhibiting platelet activation through its reduction to NO by interacting with deoxyhemoglobin. These findings strongly support the importance of hemoglobin itself as the production of NO from nitrite in the mammalian vasculature. These results may also be useful to understand the differences between arterial and venous thrombosis. Platelets are known to be predominantly involved in arterial thrombosis rather than venous thrombus formation in which trapped erythrocytes greatly contribute due to low pressure in vein. However, our results indicate that platelet inhibition by nitrite might play a critical role in regulating venous thrombosis since deoxygenated erythrocytes in relatively hypoxic veins can convert nitrite to NO and thus control platelet function and eventually blood flow. This is a plausible mechanism that explains how normal physiology of circulation regulates blood flow through the interaction between erythrocytes and platelets in vein. This proposed mechanism might support a variety of clinical entities including recent reports that show an important role of aspirin in venous thromboembolism prophylaxis [31], [32]. Thus, our results underscore the robust pathologic roles of platelets in venous clot formation which were not as much appreciated as in arterial clot formation. Replenishing nitrite ions through nitrate-rich diets or direct nitrite infusion might be useful to prevent venous thromboembolism via regulating platelet reactivity.
